# Serum Markers of Bone Turnover and Bone Remodeling in Children with Noonan Syndrome: Genotype-Phenotype Correlation

**DOI:** 10.3390/genes16060668

**Published:** 2025-05-30

**Authors:** Mariangela Chiarito, Ilaria Farella, Crescenza Lattanzio, Rossella Vitale, Flavia Urbano, Pietro Guida, Laura Piacente, Paola Muggeo, Maria Felicia Faienza

**Affiliations:** 1 Giovanni XXIII Pediatric Hospital, University of Bari “A. Moro”, 70124 Bari, Italy; mariangelachiarito@gmail.com (M.C.); c.lattanzio8@studenti.uniba.it (C.L.); r.vitale7@studenti.uniba.it (R.V.); flavia.urbano84@gmail.com (F.U.); 2 Department of Medicine and Surgery, LUM University, 70010 Casamassima, Italy; farella@lum.it; 3 Neonatology Department, Regional General Hospital “F. Miulli”, 70021 Bari, Italy; p.guida@miulli.it; 4 Pediatric Unit, Department of Precision and Regenerative Medicine and Ionian Area, University “A. Moro”, 70124 Bari, Italy; laura.piacente@uniba.it; 5 Department of Pediatric Oncology and Hematology, University Hospital of Policlinic, 70124 Bari, Italy; paola.muggeo@gmail.com

**Keywords:** Noonan syndrome, RAS/MAPK pathway, bone turnover markers, bone remodeling markers, bone mineral density

## Abstract

Noonan syndrome (NS) is a genetic disorder characterized by distinctive craniofacial and skeletal features, short stature, mild to moderate developmental impairment, and multisystem involvement, notably affecting the cardiovascular, musculoskeletal, and endocrine systems. Although abnormalities of the bone matrix, as well as osteopenia and osteoporosis, are well recognized in individuals with NS and other RASopathies, the specific impact of RAS/MAPK pathway dysregulation on bone health remains poorly understood. **Objectives:** The aim of this study was to evaluate bone turnover and bone remodeling markers in a cohort of children with NS, to gain further insights into the bone status of these patients. **Methods:** In this cross-sectional, case-control study, we analyzed 28 children (20 males) with a molecular diagnosis of NS and 35 healthy subjects (21 males), matched by age and sex. We assessed markers of bone metabolism and bone turnover (calcium, phosphate, PTH, 25(OH)-vitamin D, osteocalcin, procollagen I N-propeptide-P1NP, bone alkaline phosphatase-BALP, C-telopeptides of type I collagen-CTX) and bone remodeling (RANKL, OPG, and sclerostin). Bone mineralization was measured at the lumbar spine (L2–L4) using dual-energy X-ray absorptiometry (DEXA). **Results:** Serum CTX levels were significantly higher in NS patients compared to controls (1.8 ± 0.7 vs. 1.3 ± 0.5 ng/mL, *p* = 0.0004). RANKL levels were higher in NS patients, although the difference did not reach statistical significance. No significant differences were found for OPG, sclerostin, or other markers of bone metabolism between patients and controls. **Conclusions:** Children with NS exhibit increased bone resorption, as indicated by elevated CTX levels, suggesting a potential imbalance in bone remodeling processes. Further studies are warranted to better define the impact of RAS/MAPK pathway dysregulation on bone health in this population.

## 1. Introduction

Noonan syndrome (NS) is a genetic disorder with a prevalence ranging from 1 in 1000 to 1 in 2500 live births. It is characterized by short stature, distinctive facial and skeletal dysmorphisms (e.g., hypertelorism), down-slanting palpebral fissures, high arched palate, low set posteriorly rotated ears, ptosis, pectus excavatum or carinatum, scoliosis, mild to moderate developmental delay, and a heterogeneous pattern of abnormalities involving the cardiovascular, musculoskeletal, and endocrine systems [1,2,3].

NS belongs to the RASopathies, a group of conditions with overlapping features including cardio-facio-cutaneous syndrome, Costello syndrome, LEOPARD syndrome, Mazzanti syndrome, Legius syndrome, Neurofibromatosis–Noonan syndrome, and an increasing number of related disorders [4]. Germline mutations in genes encoding for components of the RAS/MAPK (mitogen-activated protein kinase) signaling pathway are responsible for RASopathies. This pathway is involved in cell growth, differentiation, proliferation, migration, and apoptosis [5,6,7]. The first gene associated with NS, responsible for 50% of cases, is the Protein-tyrosine phosphatase, nonreceptor-type 11 (*PTPN11*), which encodes the Src homology-containing protein tyrosine phosphatase 2 (SHP2) involved in several intracellular signals [8].

Several functionally related genes, such as *SOS1, RAF1*, *RIT1*, *KRAS*, *NRAS*, *BRAF*, *SOS2*, *SHOC2*, *LZTR1*, and others, have been associated with the pathogenesis of NS; thus, the causal mutations remain unidentified in about 10–20% of patients [9,10].

This genetic heterogeneity reflects the complexity of NS, which has various clinical manifestations that can differ widely between affected individuals.

Mutations associated with NS, particularly in the *PTPN11* gene, have a direct impact on bone development and contribute to abnormal bone growth [11]. Short stature is one of the main features of NS. Growth retardation usually occurs during the first year of life and becomes increasingly evident due to delayed puberty and the reduction in the pubertal growth spurt [12]. A decrease in bone mass, which correlates with decreased muscle mass and low IGF-1 serum levels, has been observed in children with NS [13]. An alteration of bone quality assessed using phalangeal quantitative ultrasound (QUS), in the absence of fracture risk, has also been reported in NS children [14]. In addition, reduced physical activity, hypotonia, reduced sun exposure, and low 25-OH vitamin D serum levels can increase the risk of osteopenia or osteoporosis in subjects with RASopathies.

In adult NS patients, a progression towards marked demineralization and vertebral fractures has been observed, despite treatment with bisphosphonates, teriparatide, and denosumab [15].

However, the effects of RAS/MAPK pathway dysregulation on bone health in subjects affected with NS and other RASopathies remain insufficiently explored, although alterations in bone matrix, osteopenia, and osteoporosis have been documented [11,13,16]. In addition, data on bone turnover markers in this group of diseases are few and conflicting [11,13,14].

Bone tissue is characterized by continuous remodeling in which resorption is followed by formation. The cells that regulate bone remodeling are the osteoclasts, responsible for the resorption of the bone matrix, and the osteoblasts, responsible for the synthesis of the matrix components. Bone turnover markers reflect bone cell activity. In particular, bone alkaline phosphatase (BALP), osteocalcin (OC), and procollagen I N-propeptide (P1NP) reflect the osteoblast activity, while the production of fragments of the degradation of type I collagen (N- and C-telopeptides of type I collagen- NTX and CTX) reflects osteoclast activity [17,18].

In addition, the balance of bone remodeling is regulated mainly by the nuclear factor kappa-B ligand (RANKL)/RANK/osteoprotegerin (OPG) and Wnt/β-catenin pathways, which control osteoclastogenesis and osteoblastogenesis, respectively [19,20]. Furthermore, the pro-osteoblastogenic activity of the Wnt/β-catenin pathway can be inhibited by sclerostin and Dickkopf-1 (DKK-1). Altered bone remodeling has been shown to cause bone impairment in many congenital and acquired pediatric diseases [21].

This study aimed to evaluate bone turnover and bone remodeling markers in a cohort of children with NS by assessing the serum levels of BALP, OC, P1NP, CTX, RANKL, OPG, and sclerostin to gain insights into the bone status of these patients.

In addition, we sought a possible correlation between the type of genetic mutation and bone remodeling status in these patients to identify those at risk of osteoporosis early in life.

## 2. Materials and Methods

The study group included 28 children (20 males) with a molecular diagnosis of NS, mean age at recruitment of 10.5 ± 6.07. years, who were followed at the Endocrinology and Rare Endocrine Diseases Clinic of the University of Bari, Italy.

The control group comprised 35 healthy subjects (21 males), pair matched by age (8.6 ± 4.9), attending our Pediatric Clinic for minor trauma (first aid) or allergology screening. None of them had received a bone injury. The study’s exclusion criteria for both patients and controls were the chronic use of drugs that interfere with bone metabolism (steroids, proton pump inhibitors, antiepileptics) and any immune diseases.

The study protocol was approved by the Local Ethics Committee. The children’s parents or guardians provided written informed consent. All procedures were carried out in accordance with the guidelines of the Helsinki Declaration on Human Experimentation.

### 2.1. Clinical Data

Clinical features of the NS patients, including age, sex, type of gene mutation, and information about recombinant human GH (rhGH) treatment (in progress, suspended, or never started), were collected. Anthropometric parameters, including height (H) with Harpenden stadiometer and weight (W), were assessed for every patient and were compared with the standard growth charts for the Italian population, and were expressed as a standard deviation score (SDS) [22]. Body mass index (BMI) was calculated as the ratio of weight/height2. Pubertal stages were assessed using the Tanner method [23].

### 2.2. Biochemical Assessments of Bone Metabolism Markers

After an overnight fast, venous peripheral blood samples were collected from patients and controls. The samples were promptly centrifuged and immediately frozen at −80 °C until analysis.

Calcium and phosphate levels were measured using the spectrophotometric method. Parathyroid hormone (PTH) and 25-OH vitamin D were assessed using immunological tests based on the principle of chemiluminescence (Liaison assay; DiaSorin, Stillwater, MN, USA).

Serum OC was assessed using an enzyme immunoassay (Immunodiagnostic Systems Ltd., 10 Didct Way, Boldon Business Park, Boldon, Tyne and Wear, UK, NE35 9PD) with an analytical sensitivity of 2 ng/mL and a linear range of 2–200 ng/mL. Serum P1NP was measured using an enzyme immunoassay (Immunodiagnostic Systems Ltd., 10 Didct Way, Boldon Business Park, Boldon, Tyne and Wear, UK, NE35 9PD) with an analytical sensitivity of 2 ng/mL and a linear range of 2–230 ng/mL. BALP was measured using an enzyme immunoassay (Immunodiagnostic Systems Ltd., 10 Didct Way, Boldon Business Park, Boldon, Tyne and Wear, UK, NE35 9PD) with an analytical sensitivity of 1 μg/L and a linear range of 1–75 μg/L. CTX was measured using an enzyme immunoassay (Immunodiagnostic Systems Ltd., 10 Didct Way, Boldon Business Park, Boldon, Tyne and Wear, UK, NE35 9PD) with an analytical sensitivity of 0.033 ng/mL and a linear range of 0.033–6000 ng/mL.

### 2.3. Bone Remodeling Cytokines Assessment

Sclerostin, RANKL, and OPG were assessed using ELISA (R&D Systems, Minneapolis, MN, USA; Biomedica Medizinprodukte, GmbH & Co KG, Vienna, Austria). The specific characteristics of each ELISA kit are as follows: sclerostin: assay range 0–240 pmol/L with intra-assay CV ≤ 7% and inter-assay CV ≤ 10%; RANKL: assay range 0–2 pmol/L with intra-assay CV ≤ 4% and inter-assay CV ≤ 3%; OPG: assay range 0–20 pmol/L with intra-assay CV ≤ 3% and inter-assay CV ≤ 5%.

Table 1 summarizes the role of the bone turnover markers analyzed in this study.

### 2.4. Bone Mineral Measurements

Bone mineralization was measured at the lumbar spine (L2–L4) using the dual-energy X-ray absorptiometry (DEXA) equipment at our center (ACN Unigamma X-ray Plus; L’ACN Scientific Laboratories; Hologic DiscoveryWi; Lunar iDXA, GE Healthcare) and was converted to Z-scores in relation to an age- and sex-matched normal population.

### 2.5. Statistical Analysis

Results are reported as means ± standard deviation. The Kolmogorov–Smirnov test was used to determine the normality of the parameters’ distribution. In parameters with a normal distribution, mean values were compared using the unpaired Student *t*-test, whereas linear correlations were evaluated using the Pearson correlation coefficient. The significance of parameters with a skewed distribution was assessed using the Mann–Whitney test and Spearman correlation coefficient. Variables with a non-normal distribution were expressed as medians and interquartile ranges (IQRs) and were compared using the Kruskal–Wallis test. The Statistical Package for the Social Sciences for Windows, version 27.0 (SPSS Inc., Chicago, IL, USA) was utilized for statistical analyses.

## 3. Results

### 3.1. Patients

Table 2 reports the clinical characteristics of the study population. Both groups were comparable in terms of age, sex distribution, BMI, and BMI-SDS. NS patients exhibited significantly lower height-SDS and weight-SDS compared to controls. Height and weight in absolute values were lower in NS patients, although the differences did not reach statistical significance. The mean bone age (BA) of NS subjects was 10.42 ± 4.7 years. No significant difference was observed in pubertal stage distribution between the two groups (Table 2).

**Figure 1:**
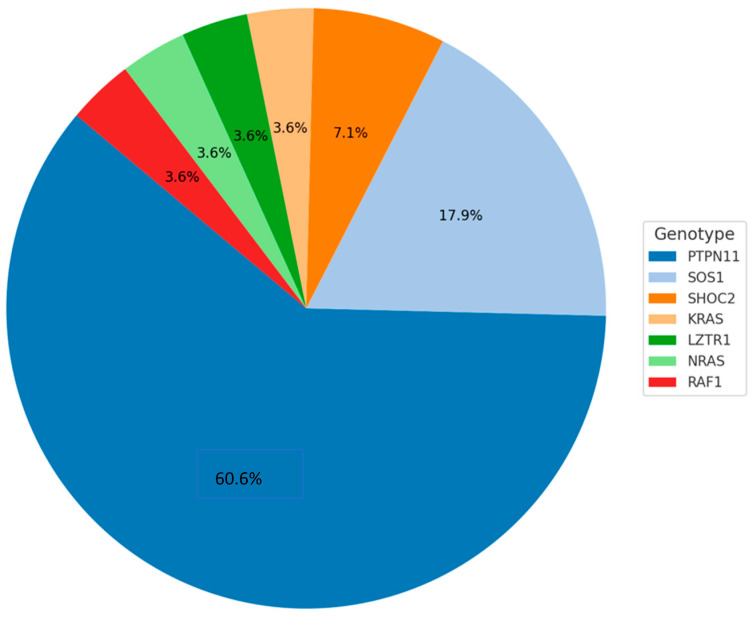
Genotype distribution in NS population.

The genetic characteristics of the NS population are shown in Figure 1.

Among them, 17 subjects (60.7%) harbored PTPN11 gene mutations, while 11 (39%) had the following gene mutations: 5 SOS1, 2 SHOC2, 1 KRAS, 1 LZTR1, 1 NRAS, and 1 RAF1—all cases were de novo mutations (Figure 1). Among the NS subjects, five had been treated with rhGH, reaching a mean final height of −3.72 ± 0.91 SDS, while two subjects were still receiving rhGH therapy at the time of recruitment. Congenital heart disease was present in 64.2% of NS subjects (14 pulmonary valve stenosis, three obstructive cardiomyopathies).

### 3.2. Bone Turnover and Bone Remodeling Markers

Table 3 shows the bone turnover and bone remodeling markers in the study population.

No difference was found between the two groups for serum levels of calcium, phosphate, PTH, OC, P1NP, or BALP. Mean values of 25-OH vitamin D in NS subjects were 26.7 ± 13.3 mg/mL; in particular, eight subjects had normal values (40.2 ± 11.1 ng/mL), 11 had insufficient values (24.5 ± 2.4 ng/mL), and nine were deficient (14.5 ± 4.6 ng/mL). NS patients had significantly higher serum CTX levels than controls (1.8 ± 0.7 vs. 1.3 ± 0.5 ng/mL, *p* = 0.0004). For bone remodeling cytokines, RANKL levels were higher in NS patients compared to controls, but this difference was not statistically significant. There were no significant differences in OPG and sclerostin levels between the two groups.

### 3.3. Bone Mineral Density Assessment

The cohort of subjects affected with NS exhibited a median bone mineral density Z-score (BMD Z score) at the lumbar spine (L1–L4) of −1.3. Specifically, 10 subjects had values below −2 Z-score (range: −4.3 to 0.79). Among these subjects, six had a PTPN11 mutation, two a SHOC2 mutation, one a KRAS mutation, and one a SOS1 mutation. Interestingly, the two subjects with a SHOC2 mutation had the most compromised BMD values (−4.3 and −2.6 Z-score, respectively). No subjects experienced fractures. A negative correlation was found between BMD Z-score and chronological age (*r* = −0.607; *p* = 0.002), whereas positive correlations were observed between BMD Z-score and phosphate levels (*r* = 0.005; *p* = 0.006), height SDS (*r* = 0.734; *p* < 0.001), BALP (*r* = 0.640; *p* = 0.001), and P1NP (*r* = 0.464; *p* = 0.034).

### 3.4. Bone Status According to the Genotype

To explore a possible genotype–phenotype correlation, we divided the NS cohort into two groups according to the presence or absence of PTPN11 gene mutations: (PTPN11+ and PTPN11-). No significant differences in age, pubertal stage, bone age, or anthropometric measures (height-SDS, weight-SDS, BMI-SDS) were observed between the two groups (Table 4). Additionally, bone turnover markers (OC, BALP, P1NP) showed no statistically significant differences between the two groups. CTX was slightly increased in PTPN11+ subjects, even if the difference was not statistically significant.

No difference in BMD-Z scores was found between rhGH-treated and untreated subjects. A significant difference was observed only in phosphate levels, with lower levels in GH-treated children (4.33 ± 0.5 mg/dL) compared to untreated children (4.80 ± 0.38 mg/dL, *p* = 0.02).

## 4. Discussion

Osteopenia and osteoporosis have been reported in NS and other RASopathies, suggesting a common pathogenesis [16]. Other comorbidities such as neurological impairment, intellectual disability, reduced physical activity, and hypotonia could affect bone health in NS subjects [24,25]. However, the mechanisms underlying reduced bone mineralization in the RASopathies are still poorly understood.

In this study, we assessed bone turnover and bone remodeling markers in a cohort of NS subjects, with the aim of investigating the bone status associated with this condition and correlating it to the genotype.

Our data demonstrated higher serum levels of CTX—a marker of bone resorption—than controls, in association with an increase in serum levels of RANKL—an osteoclastogenic cytokine—even if this increase did not reach statistical significance compared to controls. We found no differences between patients and controls with respect to sclerostin, an inhibitor of osteoblast activity, and bone metabolism parameters such as calcium, phosphate, PTH, and 25-OH vitamin D. Our data are in agreement with previous reports showing normal calcium, phosphate, PTH, 25-OH Vitamin D, and alkaline phosphatase (ALP) serum levels, and higher levels of carboxy-terminal collagen crosslinks in NS children compared to controls [11,13,14].

Markers of bone resorption identified using pyridinium crosslink analysis were also elevated in subjects affected with RASopathies compared to matched controls [26]. In our cohort of NS subjects, CTX levels were also slightly increased in PTPN11+ subjects, even if the difference was not statistically significant.

The RAS/MAPK regulates the cell cycle, differentiation, growth, and cell senescence. Specifically, SHP2 controls the ERK1/2 pathway and promotes the terminal differentiation of chondrocytes [27,28]. Inactivation of ERK1/2 in the growth plate causes chondrocytes to undergo hypertrophy prematurely and delays the replacement of cartilage with bone [29,30]. Additionally, it has been demonstrated that SHP2-deficient mice increase osteoclast activity, possibly due to increased RANKL expression in osteoblasts and chondrocytes [31].

Our data showed a reduction in BMD, particularly pronounced in subjects with SHOC2 gene mutations. These data align with Delagrange et al.’s finding of reduced axial and appendicular bone mass in a cohort of 35 NS children compared to the general population, as well as an increase in CTX serum levels [13].

Baldassarre et al. found reduced QUS measurements in 25% of NS cases, although bone metabolism markers were within the normal range [14]. Choudhry et al. documented a reduced BMD in 6 of 12 NS individuals, without any alterations in bone metabolism markers [11].

In our cohort of NS patients, we did not find a genotype–phenotype correlation for bone parameters. In addition, no difference was found in subjects treated with rhGh compared to untreated ones. In a previous study, Noordam et al. found a slight increase in bone cortical after 2 years of rhGH treatment [32].

In conclusion, despite the limited number of subjects and observational design, this is the first study to evaluate bone turnover markers, bone remodeling markers, and instrumental parameters of bone mineralization in a cohort of children with NS. The data that emerged indicate that alterations of the RAS/MAPK signaling pathway and the SHP2 protein are related to an increase in osteoclastic activity and, therefore, bone resorption, which over time could explain the tendency towards osteopenia/osteoporosis in these subjects. Future longitudinal studies in larger cohorts are needed to understand which subjects are more predisposed to skeletal demineralization and at what stage of life, and whether rhGH treatment started in the first years of life—in addition to improving height outcomes—can also maintain the balance between osteoblasts and osteoclasts, thus preserve bone health.

## Figures and Tables

**Table 1:** Main markers of bone metabolism and their functions.

Function	Marker	Full Name	Biological Role
Bone Formation	BALP	Bone-specific Alkaline Phosphatase	Enzyme involved in bone matrix mineralization
	P1NP	Procollagen type 1 N-terminal propeptide	Released during type I collagen synthesis, indicator of osteoblast activity
	Sclerostin	Sclerostin	Wnt -signaling pathway inhibitor, thus reducing bone formation
Bone Resorption	CTX	C-terminal telopeptide of type I collagen	Released during collagen degradation, marker of bone resorption.
	RANKL	Receptor Activator of Nuclear Factor κB Ligand	Stimulates osteoclast differentiation and activation
	OPG	Osteoprotegerin	Decoy receptor that binds RANKL, inhibiting bone resorption

**Table 2:** Baseline characteristics of enrolled subjects.

	NS (N = 28)	Controls (N = 35)	*p* Value
Sex (M/F)	20/8	21/14	0.3
Age (yr)	10.49 ± 6.02	8.6 ± 4.86	0.2
Height (cm)	123.26 ± 23.44	132.4 ± 19.8	0.09
Height (SDS)	−2.08 ± 1.24	−0.12 ± 0.91	0.0003
Weight (Kg)	27.30 ± 12.44	32.8 ± 11.1	0.07
Weight (SDS)	−1.79 ± 1.38	0.05 ± 1.03	0.0002
BMI (Kg/m^2^)	17.0 ± 2.92	17.9 ± 2.4	0.4
BMI (SDS)	−0.75 ± 1.24	0.11 ± 1.02	0.005
Pre-pubertal n (%)	18 (64.2)	26 (74.3)	0.4

Legend: Data are expressed as n (%) or mean ± standard deviation (SD). Significance levels: *p* < 0.05 (Student’s *t*-test or Chi-square test, as appropriate). BMI, body mass index; BMI-SDS, body mass index standard deviation score; Height-SDS, height standard deviation score; Kg, kilograms; yr, years.

**Table 3:** Markers of bone turnover and bone remodeling in the study population.

Variable	NS Subjects (*n* = 28)	Controls (*n* = 35)	*p*
Osteocalcin (ng/mL)	73.8 ± 34.7	82.0 ± 31.2	0.32 ^a^
P1NP (ng/mL)	610.8 ± 242.5	690.5 ± 333.1	0.32 ^a^
BALP (ug/L)	73.5 (53.9–95.4)	68.0 (60.8–76.3)	0.27 ^b^
CTX (ng/mL)	1.8 ± 0.7	1.3±0.4	0.001 ^a^
RANKL (pmol/L)	2582.0 (1091.7–9105.7)	1458.0 (342.0–6358.0)	0.09 ^b^
OPG (pmol/L)	3.9 ± 1.7	3.6 ± 1.3	0.44 ^a^
**RANKL/OPG ratio**	**9000.0 ± 3390.3**	**1570.5 ± 3092.1**	**0.134**
Sclerostin (pmol/L)	17.0 (16.0–19.5)	17.6 (15.2–19.7)	0.93 ^b^

Legend: Data are presented as actual numbers (%) for proportions, mean + SD for normally distributed variables, and median with interquartile range for non-normally distributed variables. NS: Noonan syndrome; P1NP: procollagen type I N-terminal propeptide; BALP: bone-specific total alkaline phosphatase; CTX: C-terminal telopeptide cross-links of type I collagen; RANKL: soluble receptor activator of nuclear factor kappa-B ligand; OPG: osteoprotegerin. ^a^ Student *t*-test, values are expressed as mean ± SD; ^b^ Mann–Whitney U test, values are expressed as median and interquartile range.

**Table 4:** Clinical and biochemical characteristics according to genotype.

Variable	PTPN11+ (*n* = 17)	PTPN11− (*n* = 11)	*p*-Value
Age (years)	10 ± 5	11 ± 8	0.972
Male (%)	13 (76%)	7 (64%)	0.671
Height SDS	−2.1 ± 1.2	−2.1 ± 1.4	0.995
Weight SDS	−1.8 ± 1.2	−1.7 ± 1.7	0.891
BMI SDS	−0.8±1.2	−0.7 ± 1.4	0.906
Calcium (mg/dL)	9.81 ± 0.47	9.88 ± 0.43	0.615
Phosphate (mg/dL)	4.71 ± 0.52	4.64 ± 0.41	0.669
PTH (pg/mL)	28 ± 12	33 ± 12	0.311
25-OH Vitamin D (ng/mL)	26 ± 13	28 ± 12	0.556
Osteocalcin (ng/mL)	87 ± 37	73 ± 27	0.710
P1NP (ng/mL)	634 ± 270	554 ± 162	0.759
BALP (ug/L)	72 ± 35	57 ± 23	0.347
CTX (ng/mL)	1.96 ± 0.74	1.74 ± 0.81	0.470
RANKL (pmol/L)	6780 ± 8492	5422 ± 5647	0.443
OPG (pmol/L)	4.0 ± 1.6	3.8 ± 2.0	0.978
BMD (Z-score)	−1.0 ± 1.1	−1.8 ± 1.4	0.1

Legend: Data are presented as actual numbers (%) for proportions, mean ± SD for normally distributed variables, and median with interquartile range for non-normally distributed variables. P1NP: procollagen type I N-terminal propeptide; BALP: bone-specific total alkaline phosphatase; CTX: C-terminal telopeptide cross-links of type I collagen; RANKL: soluble receptor activator of nuclear factor kappa-B ligand; OPG: osteoprotegerin. BMD: bone mineral density.

## Data Availability

The original contributions presented in the study are included in the article, further inquiries can be directed to the corresponding author.

## Abbreviations

The following abbreviations are used in this manuscript:

BALP	Bone-specific alkaline phosphatase
BMD	Bone mineral density
BMI	Body mass index
BMI-SDS	Body mass index standard deviation score
CTX	C-terminal telopeptide cross-links of type I collagen
DEXA	Dual-energy X-ray absorptiometry
GH	Growth hormone
LZTR1	Leucine zipper-like transcription regulator 1
NS	Noonan syndrome
OC	Osteocalcin
OPG	Osteoprotegerin
P1NP	Procollagen type I N-terminal propeptide
PTH	Parathyroid hormone
PTPN11	Protein tyrosine phosphatase non-receptor type 11
RAS	Rat sarcoma viral oncogene homolog
RASopathies	Group of genetic disorders involving the RAS/MAPK pathway
RANKL	Receptor activator of nuclear factor kappa-B ligand
rhGH	Recombinant human growth hormone
SHOC2	Leucine-rich repeat scaffold protein
SHP2	Src homology-containing protein tyrosine phosphatase 2
SOS1	Son of sevenless homolog 1
SDS	Standard deviation score
SPSS	Statistical Package for the Social Sciences

## References

[1] Roberts A.E., Allanson J.E., Tartaglia M., Gelb B.D. (2013). Noonan syndrome. Lancet.

[2] Libraro A., D’Ascanio V., Cappa M., Chiarito M., Digilio M.C., Einaudi S., Grandone A., Maghnie M., Mazzanti L., Mussa A. (2021). Growth in children with noonan syndrome and effects of growth hormone treatment on adult height. Front. Endocrinol..

[3] Faienza M.F., Meliota G., Mentino D., Ficarella R., Gentile M., Vairo U., D’amato G. (2024). Cardiac phenotype and gene mutations in RASopathies. Genes.

[4] Zenker M. (2022). Clinical overview on RASopathies. Am. J. Med. Genet. Pt. C.

[5] Aoki Y., Niihori T., Inoue S., Matsubara Y. (2016). Recent advances in RASopathies. J. Hum. Genet..

[6] Simanshu D.K., Nissley D.V., McCormick F. (2017). RAS proteins and their regulators in human disease. Cell.

[7] Tartaglia M., Gelb B.D. (2010). Disorders of dysregulated signal traffic through the RAS-MAPK pathway: Phenotypic spectrum and molecular mechanisms. Ann. N. Y. Acad. Sci..

[8] Tartaglia M., Mehler E.L., Goldberg R., Zampino G., Brunner H.G., Kremer H., Van Der Burgt I., Crosby A.H., Ion A., Jeffery S. (2001). Mutations in PTPN11, encoding the protein tyrosine phosphatase SHP-2, cause noonan syndrome. Nat. Genet..

[9] Johnston J.J., Van Der Smagt J.J., Rosenfeld J.A., Pagnamenta A.T., Alswaid A., Baker E.H., Blair E., Borck G., Brinkmann J., Craigen W. (2018). Autosomal recessive noonan syndrome associated with biallelic LZTR1 variants. Genet. Med..

[10] Tartaglia M., Aoki Y., Gelb B.D. (2022). The molecular genetics of RASopathies : An update on novel disease genes and new disorders. Am. J. Med. Genet. Pt. C.

[11] Choudhry K.S., Grover M., Tran A.A., O’Brian Smith E., Ellis K.J., Lee B.H. (2012). Decreased bone mineralization in children with noonan syndrome: Another consequence of dysregulated RAS MAPKinase pathway?. Mol. Genet. Metab..

[12] Malaquias A.C., Brasil A.S., Pereira A.C., Arnhold I.J.P., Mendonca B.B., Bertola D.R., Jorge A.A.L. (2012). growth standards of patients with noonan and noonan-like syndromes with mutations in the RAS/MAPK pathway. Am. J. Med. Genet. Pt. A.

[13] Delagrange M., Rousseau V., Cessans C., Pienkowski C., Oliver I., Jouret B., Cartault A., Diene G., Tauber M., Salles J.-P. (2021). Low bone mass in noonan syndrome children correlates with decreased muscle mass and low IGF-1 levels. Bone.

[14] Baldassarre G., Mussa A., Carli D., Molinatto C., Ferrero G.B. (2017). Constitutional bone impairment in noonan syndrome. Am. J. Med. Genet. Pt. A.

[15] Reyad M., Murad M.R., Sobhy A., Hassan A.M., Nassar O., Hassan A. (2025). Noonan syndrome and osteoporosis: A comprehensive case study and literature review. ASIDE Case Rep..

[16] Stevenson D.A., Viscogliosi G., Leoni C. (2022). Bone health in RASopathies. Am. J. Med. Genet. Pt. C.

[17] Brescia V., Lovero R., Fontana A., Zerlotin R., Colucci S.C., Grano M., Cazzolla A.P., Di Serio F., Crincoli V., Faienza M.F. (2024). Reference intervals (RIs) of the bone turnover markers (BTMs) in children and adolescents: A proposal for effective use. Biomedicines.

[18] D’Amato G., Brescia V., Fontana A., Natale M.P., Lovero R., Varraso L., Di Serio F., Simonetti S., Muggeo P., Faienza M.F. (2024). Biomarkers and biochemical indicators to evaluate bone metabolism in preterm neonates. Biomedicines.

[19] Krishnan V. (2006). Regulation of bone mass by Wnt signaling. J. Clin. Investig..

[20] Theill L.E., Boyle W.J., Penninger J.M. (2002). RANK-L and RANK: T cells, bone loss, and mammalian evolution. Annu. Rev. Immunol..

[21] Brunetti G., D’Amato G., Chiarito M., Tullo A., Colaianni G., Colucci S., Grano M., Faienza M.F. (2019). An update on the role of RANKL–RANK/osteoprotegerin and WNT-ß-catenin signaling pathways in pediatric diseases. World J. Pediatr..

[22] Cacciari E., Milani S., Balsamo A., Dammacco F., De Luca F., Chiarelli F., Pasquino A., Tonini G., Vanelli M. (2002). Italian cross-sectional growth charts for height, weight and BMI (6–20 y). Eur. J. Clin. Nutr..

[23] Tanner J.M., Whitehouse R.H. (1976). Clinical longitudinal standards for height, weight, height velocity, weight velocity, and stages of puberty. Arch. Dis. Child..

[24] Johnson B., Goldberg-Strassler D., Gripp K., Thacker M., Leoni C., Stevenson D. (2015). Function and disability in children with costello syndrome and cardiofaciocutaneous syndrome. Am. J. Med. Genet. Pt. A.

[25] Leoni C., Triumbari E.K.A., Vollono C., Onesimo R., Podagrosi M., Giorgio V., Kuczynska E., Veltri S., Tartaglia M., Zampino G. (2019). Pain in individuals with RASopathies: Prevalence and clinical characterization in a sample of 80 affected patients. Am. J. Med. Genet. Pt. A.

[26] Stevenson D., Schwarz E., Carey J., Viskochil D., Hanson H., Bauer S., Cindy Weng H.-Y., Greene T., Reinker K., Swensen J. (2011). Bone resorption in syndromes of the Ras/MAPK pathway. Clin. Genet..

[27] Kim H.K., Feng G.-S., Chen D., King P.D., Kamiya N. (2014). Targeted disruption of *Shp2* in chondrocytes leads to metachondromatosis with multiple cartilaginous protrusions. J. Bone Miner. Res..

[28] Yang W., Wang J., Moore D.C., Liang H., Dooner M., Wu Q., Terek R., Chen Q., Ehrlich M.G., Quesenberry P.J. (2013). Ptpn11 deletion in a novel progenitor causes metachondromatosis by inducing hedgehog signalling. Nature.

[29] Matsushita T., Chan Y.Y., Kawanami A., Balmes G., Landreth G.E., Murakami S. (2009). Extracellular signal-regulated kinase 1 (ERK1) and ERK2 play essential roles in osteoblast differentiation and in supporting osteoclastogenesis. Mol. Cell. Biol..

[30] Sebastian A., Matsushita T., Kawanami A., Mackem S., Landreth G.E., Murakami S. (2011). Genetic inactivation of ERK1 and ERK2 in chondrocytes promotes bone growth and anlarges the spinal canal. J. Orthop. Res..

[31] Wang L., Huang J., Moore D.C., Zuo C., Wu Q., Xie L., Von Der Mark K., Yuan X., Chen D., Warman M.L. (2017). SHP2 regulates the osteogenic fate of growth plate hypertrophic chondrocytes. Sci. Rep..

[32] Noordam C., Span J., Van Rijn R.R., Gomes-Jardin E., Van Kuijk C., Otten B.J. (2002). Bone mineral density and body composition in noonan’s syndrome: Effects of growth hormone treatment. J. Pediatr. Endocrinol. Metab..

